# Short-and-Long-Term Outcomes after Coronary Rotational Atherectomy in Patients Treated with Trans-Catheter Aortic Valve Implantation

**DOI:** 10.3390/jcm10010112

**Published:** 2020-12-31

**Authors:** Mattia Lunardi, Michele Pighi, Gabriele Venturi, Paolo Alberto Del Sole, Gabriele Pesarini, Andrea Mainardi, Roberto Scarsini, Valeria Ferrero, Leonardo Gottin, Flavio Ribichini

**Affiliations:** 1 Division of Cardiology, Department of Medicine, University of Verona, 37129 Verona, Italy; mattia.lunardi@outlook.com (M.L.); michele.pighi@univr.it (M.P.); gabriele.venturi.vr@gmail.com (G.V.); paoloalbertodelsole@gmail.com (P.A.D.S.); gabriele.pesarini@aovr.veneto.it (G.P.); m.a.mainardiandrea@gmail.com (A.M.); scarsini.roberto@gmail.com (R.S.); valeria.ferrero@aovr.veneto.it (V.F.); 2 Division of Anesthesiology, Department of Surgery, University of Verona, 37129 Verona, Italy; leonardo.gottin@univr.it

**Keywords:** high-risk aortic valve stenosis, trans-catheter aortic valve implantation, catheter-based coronary interventions, rotational atherectomy, percutaneous coronary interventions

## Abstract

Background. Coronary artery disease (CAD) is a common finding among patients undergoing trans-catheter aortic valve implantation (TAVI), who often present severely calcified coronary lesions. Evidence is scarce about the use of rotational atherectomy (RA) in this setting, in particular regarding long-term outcome. Methods. RA was performed on severely calcified coronary lesions concomitant with TAVI in a consecutive series of patients treated between 2010 and 2020. Immediate and long-term clinical outcomes are reported. Results. A concomitant CAD (coronary stenosis visually > 50%) was observed in 402/845 (47.6%) consecutive patients undergoing TAVI at the University Hospital of Verona. Angioplasty was performed in 104 patients (12.3%). Among these, 19 patients (18.3%, 20 coronary arteries), were treated with RA after TAVI: 10 after implantation of a balloon-expandable trans-catheter valve and 9 after a self-expandable valve. All procedures were successful. Hypotension occurred in 3 patients (15.8%), with rapid recovery after the procedure; CI-AKI (contrast-induced acute kidney injury) in 3 patients (15.8%), of which two recovered within discharge. At a median follow-up of 21.5 months (Q1–3: 6–36) event free survival was 83.3%. Only one patient suffered a target vessel failure >2 years after RA. Neither stroke nor peri-procedural infarctions were detected. Conclusions. RA concomitant with TAVI was feasible and safe in patients treated with implantation of either self-expandable, or balloon-expandable trans-catheter aortic valves. Long-term clinical events related to the coronary procedure were extremely infrequent and the survival rate at median follow-up of 21.5 months was 83.3%.

## 1. Introduction

Coronary artery disease (CAD) is a common finding among patients with severe aortic valve stenosis (AVS) candidate to Transcatheter Aortic Valve Implantation (TAVI) [1,2].

This population often present severely calcified CAD with an increased risk of intra-procedural complications and mid-term clinical events related to stent restenosis and stent thrombosis due to sub-optimal lesion preparation.

Rotational atherectomy (RA) could represent a valid aid for plaque debulking, in order to optimize the revascularization results in such cases.

Nevertheless, the implementation of RA to support percutaneous coronary intervention (PCI) after TAVI is uncommon, mostly due to apparent technical issues.

In a previous report [3] we demonstrated that RA after TAVI was feasible and safe in the short-term, either through a self-expandable or a balloon-expandable trans-catheter aortic valve. However, evidence is scarce about the long-term impact of such an approach on clinical outcomes, since available data focuses on procedural feasibility and in-hospital/short term outcomes, up to 30-day.

The aim of the present study was to investigate the clinical efficacy at long-term follow up of RA-assisted PCI after TAVI. Moreover, the study sought to enhance the analysis of RA feasibility in this scenario, presenting a larger single-centre series.

## 2. Methods

### 2.1. Study Population

This is a retrospective analysis of prospectively collected data of patients presenting symptomatic severe AVS undergoing TAVI and RA-assisted PCI at the University Hospital of Verona, Italy, from March 2010 to August 2020.

Patients were addressed to TAVI when presenting increased surgical risk as predicted by STS score >4, and in all cases after Heart Team discussion [4].

All patients provided their written informed consent to be enrolled in the local registry (Valvular Verona Registry, CESC *n* = 1918), approved by the ethical review board of the University of Verona.

### 2.2. TAVI Procedure

TAVI were performed either by the percutaneous trans-femoral or trans-apical approach when patients presented severe ilio-femoral vascular disease, aorto-femoral bypass or occluded aorta. The Edwards SAPIEN-XT, S3 or ULTRA (Edwards Lifesciences, Irvine, CA, USA), the Medtronic CoreValve, Evolut-R or Pro (Medtronic Inc., Minneapolis, MN, USA) were used, according to the anatomic characteristics of the valve morphology as analysed at the CT-scan.

### 2.3. Percutaneous Coronary Interventions

PCI were performed in case of critical stenosis according to angiographic evaluation. Critical stenosis was defined by % Diameter Stenosis (DS) > 50 for LM and > 70 otherwise. For borderline lesions, functional evaluation was performed through Fractional Flow Reserve (FFR).

The presence of the following lesion characteristics indicated RA use:

- Ostial and heavily calcified lesions that cannot be dilated by using balloon angioplasty;
- Severe calcified stenosis where a stent cannot be delivered to its target.

**Figure 1:**
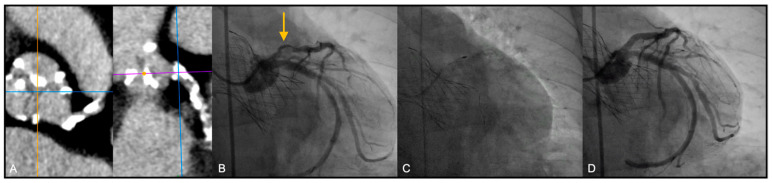
Rotational atherectomy (RA) following Medtronic CoreValve deployment by Trans-Femoral approach. (**A**) Pre-TAVI CT views showing severe calcification of LAD; (**B**) Pre RA angiographic evaluation of the lesion: proximal LAD presented severe calcified lesion (arrow); (**C**) Multiple burr (RotaPro 1.25 mm) advancements; (**D**) Final result, after stent deployment and post dilation.

In addition, in several patients, other than the angiographic evaluation of the coronary calcification, the coronary analysis of the pre-TAVI CT scan has added important information regarding the extension of the vessel calcification (see as an example in Figure 1, part A).

Last, IVUS was used to confirm the degree of vessel calcification in 4/19 cases (21.0%).

Before coronary interventions, all patients were pre-treated with a loading dose of aspirin (300 mg) and clopidogrel (300 mg).

Percutaneous radial or femoral arterial accesses were used, using either a 6Fr (radial or femoral access) or 7–8 Fr (femoral access) sheath. Conventional Judkins right or EBU left 6Fr guiding catheters were used.

Mechanical and pharmacologic support could be planned as an upfront strategy according to operator preferences based on a case-by-case evaluation, or as a bailout strategy, in case of abrupt hemodynamic impairment. As for mechanical support (MCS) Intra-aortic Balloon Pump (Arrow^®^, Teleflex, Morrisville, NY, USA) was adopted in some cases. As for pharmacologic support, Noradrenalin, Dobutamine or Dopamine could be used according to operators’ and anesthesiologists’ evaluation of the case.

After coronary stent implantation patients received standard medications, including a double anti-platelet therapy comprising aspirin 100–160 mg/day and clopidogrel 75 mg/day or ticagrelor 90 mg twice a day for at least 6 months up to 12 months. Patients treated with PCI having an indication to oral anti-coagulation had triple anti-thrombotic therapy for 1-month, oral anti-coagulation plus aspirin or clopidogrel up to 12 months, only oral anticoagulation thereafter. All patients were treated with second generation drug eluting stent (DES).

### 2.4. Rotational Atherectomy

The protocol for RA used in our Institution follows the recommendations of the European RA expert group [5].

Accordingly, a single-curve guiding catheter is preferred in most cases, due to the strong support offered (Judkins Right 4 or EBU). Then, a floppy rotawire is advanced across the lesion. In case of challenging advancement, a regular wire can be placed distally to the lesion and then switched with the rotawire using a microcatheter. Plaque debulking starts with small burs (1.25 to 1.5 mm) as an initial strategy, followed by a step-up approach to limit debris size and complications, maintaining a burr-artery ratio of <0.6. Low speeds (135,000 to 180,000 rpm) are usually sufficient to obtain adequate plaque modification. No special rotablation cocktail was used in the flush solution, just composed of saline and heparin.

Routine use of temporary PM is not recommended. However, having most of these procedures been performed immediately after the TAVI (16/19, 84.2%) a temporary pacemaker catheter was already inserted in most cases. In the 3 cases (15.8%) of RA performed after the TAVI procedure, a temporary pacemaker was not preventively implanted.

In our series, nitrates were used during PCI as in any routine coronary intervention. Please note that, performing the procedure after TAVI allows a safe use of nitrates since the aortic stenosis has been already removed.

No particular technique was used in the presence of balloon-expandable valves, which are commonly recognized as “intra-annular” and for this reason not hindering access to coronary ostia.

Conversely, the frames of self-expandable valves always interpose in front of the coronary origins. They allow however catheters >10Fr through the struts (major axis of the inflow cells is 10 mm in the Evolut-R valve, compared to 8 mm in the CoreValve type). Of note, the passage of the catheter through the diamond-shaped valve struts provides, itself, a good support to the guiding catheter even if not deeply or selectively engaged into the coronary.

In some cases, the difficulty in the advancement of Rotawire across the lesion was overcome by using a microcatheter for wires switching.

In all the cases, RA was performed after TAVI because it was assumed that any eventual ischemic complication related to the PCI (slow flow or vessel occlusion, leading to acute worsening of the left ventricular performance) would have been better tolerated after the complete release of the left ventricle pressure overload.

Moreover, the ischemia related to the rapid ventricular pacing during balloon-expandable valve implantation is very short and unlikely to create irreversible hemodynamic impairment, even before myocardial revascularization.

In addition, for patients with an estimated large ischemic burden (unprotected left main (ULM) stenosis, proximal left anterior descending (LAD) and ostial stenosis of very large dominant right coronary arteries (RCA) with impaired left ventricular function), self-expandable valves were generally preferred so that rapid ventricular pacing was not even needed.

In most cases of TF-TAVI, the femoral vascular access used to implant the valve was used to perform the RA-assisted PCI.

Among the TA-TAVI patients, RA-assisted PCI was performed through the right or left radial access in most cases.

### 2.5. Clinical Follow-Up and Adverse Clinical Events Definition

The occurrence of procedural-related complications was prospectively evaluated, as well as in-hospital adverse events and long-term clinical follow-up.

All adverse clinical events were defined according to VARC-2 recommendations [6].

Procedural complications included: death, major stroke, side branch occlusion, peri-procedural hypotension, need for inotropic (drug-based) or circulatory (mechanical) support, cardiac tamponade, life-threatening arrhythmias, conversion to cardiac surgery or major vascular complications.

The assessed in-hospital adverse events included: death, any troponin rise, peri-procedural (type 4a) myocardial infarction (MI) defined as the occurrence of new ischemic symptoms or signs in addition to elevation of cardiac biomarkers (peak value exceeding 15× as the upper reference limit for troponin or 5× for CK-MB) ≤ 72 h after the index procedure, contrast-induced acute kidney injury (CI-AKI: serum creatinine value increase >0.3 mg/dL compared to baseline within 72 h) and need for dialysis.

After discharge, clinical follow-up assessment was conducted during outpatients’ clinic visits at 1, 6, 12 and 24 months. In particular, data about occurrence of death, cardiovascular death, major stroke, acute coronary syndrome (ACS), need for urgent revascularization, target lesion failure (TLF), renal function, need for permanent pacemaker implantation and left ventricular ejection fraction we carefully assessed.

Longer term follow-up was thereafter collected yearly through telephonic interviews or clinical evaluation.

The composite event of major adverse cardiovascular and cerebrovascular events (MACCE) was defined as the occurrence of cardiac death, major stroke, ACS and TLF.

### 2.6. Endpoints

The primary endpoint was defined as the occurrence of MACCE at long-term follow-up.

The secondary endpoint was defined as the occurrence of any procedural complication related either to TAVI or RA-assisted PCI.

### 2.7. Statistical Analysis

Categorical data are presented as numbers and percentages. Continuous data are presented as means and standard deviations (SD) for normally distributed variables and as median and interquartile range (IQR) otherwise.

All the statistical analysis was performed with the use of SPSS 24.0 software (SPSS Inc. Chicago, IL, USA).

## 3. Results

Between March 2010 and August 2020, 845 patients underwent TAVI at the University Hospital of Verona. Among these, 402 patients (47.6%) presented CAD defined as a coronary stenosis >50% as to visual estimation in at least one of the main coronary arteries. In 104 (25.9%) patients. CAD was treated with PCI either as a staged procedure before or after TAVI or as a concomitant procedure.

In 19 cases (18.3% of all PCI) RA was performed as debulking technique on severe calcific lesions.

### 3.1. Baseline Characteristics

The high-risk nature of the patients population was mainly driven by the advanced age (medium age was 81.4 ± 7.2) and medium-high SYNTAX score. Mean LV function and mean eGFR were 51.4 ± 14.5% and 54.7 ± 17.8 mL/min/1.73 m^2^ respectively, 42.1% were women. Mean STS score was 5.3 ± 5.0.

Three patients (15.8%) were treated under critical conditions: one with signs of extensive ongoing myocardial ischemia, and two with refractory heart failure evolving into cardiogenic shock. All the other patients were treated under stable hemodynamic conditions.

All baseline characteristics are described in Table 1.

### 3.2. Procedural Characteristics and Outcomes

In 10 out of 19 (52.6%) patients included in the present study a balloon-expandable valve was implanted, while the remaining 9 patients (47.4%) received a self-expandable valve.

**Figure 2:**
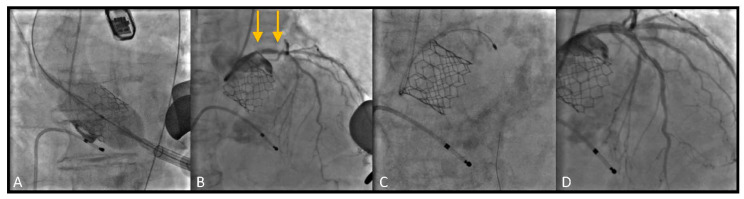
Rotational atherectomy (RA) following Edwards Sapien valve deployment by Trans-apical approach. (**A**) Balloon expandable valve (Edwards Sapien) deployment; (**B**) Pre RA angiographic evaluation of the lesion: severe calcified lesion of the ULM and ostial LAD (arrows); (**C**) Multiple Burr (RotaPro 1.5 mm) advancements; (**D**) Final result, after stent deployment and post dilation.

Most patients underwent TAVI via trans-femoral route (Figure 1). In 5 cases (26.3%) TAVI procedure was performed through a trans-apical access due to important vascular disease involving the ilio-femoral axis, not suitable for the trans-femoral TAVI delivery systems (Figure 2).

In two patients the RA-assisted PCI were performed by the radial access after TA-TAVI.

RA was performed after TAVI in all cases; concomitant with TAVI after valve deployment in 16 cases (84.2%), while in 3 cases (15.8%) it was performed as a staged procedure within one month after TAVI (3, 7 and 12 days). In one of the staged procedures, hemodynamic instability occurred during TAVI, so that the operator preferred performing RA after patient stabilization. In the other two cases, the complex coronary scenario revealed by the coronary angiography during TAVI led the operators to postpone PCI to heart team discussion and case preparation.

All procedures were performed successfully and with implantation of DES after plaque debulking with RA and balloon dilatation before stenting.

IVUS was used in all cases of left main PCI, both for pre-PCI assessment and for stent optimization.

Mean amount contrast medium used, considering the sum of TAVI and RA procedure in case of staged approach, was 210.7 ± 92.4 mL. Mean procedural time was 164.0 ± 51.1 min.

Inotropic support was used as upfront strategy in two cases (one together with mechanical IABP support), that experienced significant procedural hypotension (10.5%) restored with vasoactive drugs. Another patient (5.3%) presented unexpected hemodynamic worsening, requiring bailout inotropes infusion. In all these patients a rapid hemodynamic recovery was observed after the end of the PCI. No cases of major ventricular arrhythmias and cardiac tamponade were observed, and no patient required vascular surgery or conversion to cardiac surgery.

Complete procedural characteristics and outcomes are reported in Table 2.

### 3.3. In Hospital Outcomes

In-hospital stay mean length was 9.4 ± 10.5 days; of these 2.7 ± 5.7 in the intensive care unit.

Serum creatinine levels did rise significantly in three patients (15.8%) with two of them (10.5%) treated with TAVI and PCI in emergency conditions (cardiogenic shock and hypotension with refractory ventricular tachycardia), requiring a short cycle of renal replacement therapy. However, their renal function returned to baseline after hemodynamic stabilization.

None of the remaining patients had a significant rise of creatinine after TAVI and RA-assisted PCI, and no permanent pace-maker implantation was needed at the time of hospital discharge.

A non-significant troponin release as detected by ultra-sensitivity method (any elevation >15 ng/L) was observed in all cases between 6 and 48 h after procedure. However, no peri-procedural myocardial infarction occurred.

In Hospital complications are reported in Table 3.

### 3.4. Long Term Follow-Up

Long term follow-up was available for a total of 18 out of 19 patients, who were discharged since one patient died before discharge for acute intestinal ischemia.

Median follow-up length was 21.5 months (interquartile range Q1–Q3 = 6–36 months).

Six patients (33.3%) died at long-term follow-up: three (16.7%) for cardiac causes and 3 (16.7%) for extra-cardiac causes.

One patient (5.6%), who underwent RA on ostial RCA with stenting of proximal RCA was admitted two years after TAVI for an inferior NSTEMI with angiographic documentation of in-stent restenosis (sub-occlusion of proximal RCA). The same patient died for heart failure at the age of 93, 49 months after the index procedure. Of note, no further TLF were reported.

Three patients (16.7%) required a permanent pace-maker implantation during follow up, which occurred respectively after a period of three months, sixteen months and twenty-four months from the discharge date.

Mean eGFR and LVEF values at follow-up did not show a significant variation when compared to baseline values before the procedure.

Long-term follow-up events are reported in Table 4.

## 4. Discussion

The main findings of this study are the feasibility, the safety and the long-term clinical efficacy of RA-assisted PCI in patients with complex CAD undergoing TAVI.

To our best knowledge, this is the first study reporting the long-term outcome of combined RA and TAVI. These results support our previously reported data [3].

CAD is often observed during the pre-TAVI diagnostic examinations, in most cases as an incidental finding, and the management of coronary stenosis in this setting is not standardized.

Coronary artery bypass surgery concomitant with SAVR improve long-term survival compared to SAVR alone in patients with CAD [7] and is therefore recommended by international guidelines if any coronary stenosis >50% is present [8].

Evidences in favor of percutaneous revascularization in TAVI patients are instead contradictory [9,10] and current guidelines recommend PCI without a consistent scientific evidence (level of evidence: C) [11]. Accordingly, there is no consensus on the need for treating coronary stenosis in TAVI patients in the absence of clear signs of ischemia.

However, the rapid expansion of TAVI indications in younger patients as an alternative to SAVR is likely to expand also the indications for the treatment of the bystander CAD with a combined approach of TAVI and percutaneous myocardial revascularization. Nevertheless, patients undergoing TAVI usually present complex CAD which may need aggressive plaque debulking techniques in some cases. In these setting, RA can facilitate stent delivery and adequate stent expansion with the consequent reduction of the risk of procedural failure, stent under-expansion and potentially stent thrombosis and restenosis. Following these considerations, the number of TAVI patients needing RA is likely to increase in the next years.

In our series, RA was used in 18.3% of all the PCI procedures performed in TAVI patients, a five to ten-fold higher percentage compared to that reported in clinical practice among non-TAVI patients [5].

However, available data about RA in TAVI focuses on feasibility and short-term outcome [12,13], with no data on the long-term outcome.

Regarding the feasibility, a series of 25 patients from a Japanese multi-centre registry (OCEAN Registry) has been published: in almost all cases RA and PCI were performed in a staged fashion before TAVI [14].

We believe that such approach may lead to more potential ischemic complications related to the PCI in case of slow flow or vessel occlusion, due to the still present aortic stenosis and the related left ventricular increased oxygen demand as demonstrated by the routinely use of balloon pump, inotropic support and intra-vascular ultrasound as preventive measures in the OCEAN Registry (16% of patients required circulatory support with IABP, 40% with intravenous catecholamine) [14].

Similarly, Lippmann et al. demonstrated the feasibility of RA-facilitated PCI in a series of 29 patients with severe AS patients under consideration for TAVI. A significant pressure and heart rate drop in 24 patients (82.8%) was observed and up to 1/3 of the cases needed for inotropic support and/or temporary pacing during the procedure [15].

The potential risk of sustained hemodynamic instability during RA observed in severe aortic stenosis patients may support our strategy of undertaking complex PCI after the valve deployment. Moreover, concerns about performing TAVI in patients with underlying ischemia seems no more justifiable, since recent evidence from meta-analysis data has shown that PCI before TAVI does not improve MACE-free survival and can actually increase the risk of complications [16].

In support of this position is the observation that even in the higher risk patients with TA-TAVI, and with severe calcified CAD of the left main or the proximal LAD, balloon-expandable valves were implanted under rapid pacing before treating the CAD without relevant hemodynamic issues, and the RA-assisted PCI was performed after the TAVI without complications.

Our very first experience obtained in 2 patients showed the feasibility of performing urgent RA during the same TAVI procedure after the valve deployment [17], and a third case was performed as an emergency intervention 8 days after TAVI. Based on these encouraging results, we adopted this approach in the following cases, even in more complex cases presenting with acute ischemia, severe left main stem stenosis, impaired left ventricular contractility or even with cardiogenic shock. We adopted this strategy because we thought that possible PCI complication would have been better tolerated after the complete release of the ventricular pressure overload. Indeed, we observed no periprocedural mortality and major complications. Moreover, mechanical and pharmacological circulatory support was needed in a lower number of patients (5.3% and 15.8% respectively) compared to other series and strictly limited to patients presenting unstable hemodynamic baseline conditions (i.e., cardiogenic shock with planned use of IABP and inotropes, and one case of hypotension with refractory ventricular arrhythmias). Only one case (5.3%) required bailout use of inotropes because of sudden hypotension during the intervention. However, in all the three cases the procedure was completed successfully with no major complications observed during in-hospital stay.

Available data does not allow to tell whether RA would rather be done during the same TAVI session or as a deferred procedure; however, in the absence of complications related to the TAVI procedure, the concomitant strategy is feasible and appears reasonable since it lowers the risk for vascular complications and reduces in-hospital stay and costs in both, TF and TA-TAVI.

Regarding the possible impact of a concomitant versus staged procedure on renal function, some concerns may rise among clinicians about the safety of exposing patients to higher contrast medium dose potentially used in such complex procedures. However, our experience supports the choice of a concomitant strategy as safer in term of CI-AKI risk. Indeed, severe aortic stenosis patients undergoing staged coronary procedures before TAVI are likely to be more exposed to contrast medium damage, since cardiac output before aortic valve substitution is compromised and consequently peripheric and renal perfusion is lower than after valve replacement.

In the present manuscript, we analysed the impact of RA combined with TAVI on short-term and long-term renal function. In our series, we found an incidence of CI-AKI of 15.8%, in line with previous reports about TAVI alone procedures [18]. In two of the three cases, patients had a complete in-hospital recovery of renal function after a brief cycle of renal replacement therapy. Moreover, at a long-term follow up after TAVI and RA, renal function stabilized compared to baseline with a trend, albeit non-significant, toward higher eGFR values. This phenomenon, as previously hypothesized [19], is likely due to the hemodynamic recovery following aortic valve implantation which by increasing systemic and renal artery flow could mitigate contrast medium-related damage.

Finally, our series provide, for the first time, information about the long-term clinical outcomes after RA-assisted PCI in TAVI. The MACCE-free survival rate at a mean follow-up of 24 months was 83.3%, comparable with that of TAVI patients without CAD [20]. Of note, two out of the three cardiac deaths occurred later than 4 years after TAVI, in patients older than 90.

At long-term follow-up, coronary adverse events consisted only in one target lesion failure.

Our data should be interpreted with caution given the large experience of our group in performing RA [4,5] and the descriptive nature of the current study. Indeed, no definitive conclusions can be drawn on these bases, and this analysis should be considered hypothesis-generating to sustain further dedicated studies to validate this approach and its widespread applicability.

## 5. Study Limitations

The number of patients reported is still limited because of their high complexity and the single centre nature of the observation. RA is a technically demanding procedure: the results obtained may widely vary depending on centres’ experience.

## 6. Conclusions

RA concomitant with TAVI after valve deployment is feasible and safe in patients presenting AVS associated with calcified CAD, leading to satisfactory immediate and long-term clinical outcomes.

## Figures and Tables

**Table 1:** Baseline characteristics.

Baseline Characteristics	19 Patients
Age (years)	81.4 ± 7.2
Female, *n* (%)	8 (42.1%)
Diabetes, *n* (%)	8 (42.1%)
HTN, *n* (%)	16 (84.2%)
Peripheral artery disease, *n* (%)	11 (57.9%)
Previous cardiac surgery, *n* (%)	3 (15.8%)
NYHA III-IV class pre-TAVI, *n* (%)	15 (78.9%)
eGFR, mL/min	54.7 ± 17.8
Atrial fibrillation, *n* (%)	6 (31.6%)
LVEF, %	51.4 ± 14.5
AVA pre-TAVI, cm^2^	0.6 ± 0.4
Mean Transaortic Gradient pre-TAVI, mmHg	42.8 ± 13.5
Multivessel disease, *n* (%)	11 (57.9%)
SYNTAX score, %	23.3 ± 13.5
Logistic EuroSCORE, %	22.0 ± 16.5
STS score, %	5.3 ± 5.0

HTN: Hypertension, NYHA: New York Functional Class, eGFR: estimated Glomerular Filtration Rate, LVEF: Left ventricular Ejection Fraction, AVA: Aortic Valve Area; STS: Society of Thoracic surgery Score.

**Table 2:** Procedural characteristics and outcome.

Procedural Characteristics and Outcomes	19 Patients
Balloon expandable valve, *n* (%)	10 (52.6%)
Trans-apical approach, *n* (%)	5 (26.3%)
Procedure time, min	164.0 ± 51.05
Amount of contrast, mL	210.7 ± 92.4
Burr size, mm	1.5 ± 0.3
Death, *n* (%)	0
Major stroke, *n* (%)	0
Side branch occlusion, *n* (%)	0
Peri-procedural hypotension, *n* (%)	3 (15.8%)
Need for inotropic support, *n* (%)	3 (15.8%)
Need for circulatory support with IABP, *n* (%)	1 (5.3%)
Cardiac tamponade, *n* (%)	0
Conversion to cardiac surgery, *n* (%)	0
VT-VF, *n* (%)	0
Major vascular complications, *n* (%)	0

IABP: Intra-aortic-balloon-pump, VT-VF, Ventricular Tachycardia-Ventricular Fibrillation.

**Table 3:** In hospital complications.

In-Hospital Outcomes	19 Patients
Hospital stays, days	9.9 ± 10.5
ICU stay, days	2.7 ± 5.7
Death, *n* (%) *	1 (5.3%)
Troponin rise, *n* (%)	19 (100%)
Peri-procedural myocardial infarction, *n* (%)	0
CI-AKI, *n* (%)	3 (15.8%)
Need for dialysis, *n* (%)	2 (10.5%)

ICU: intensive Care Unit, CI-AKI: Contrast-induced acute kidney injury. * non-cardiac.

**Table 4:** Long term follow-up outcomes.

Long-Term Follow-Up	18 Patients
Death, *n* (%)	6 (33.3%)
Death from cardiac cause, *n* (%)	3 (16.7%)
Major stroke, *n* (%)	0
ACS, *n* (%)	1 (5.6%)
Need for urgent revascularization, *n* (%)	0
TLF, *n* (%)	1 (5.6%)
eGFR at follow up, mL/min	52.0 ± 17.6
PPM, *n* (%)	3 (16.7%)
Left ventricular ejection fraction, %	51.1 ± 10.8
Cumulative MACEs, *n* (%)	3 (16.7%)
Event free survival	15 (83.3%)

ACS: acute coronary syndrome, PPM: permanent pace-maker implantation, MACEs: major adverse cardiac event.

## Data Availability

The data presented in this study are available on request from the corresponding author. The data are not publicly available due to local policy regulating patients’ data sharing.

## Abbreviations

ACS	acute coronary syndrome
AVS	aortic valve stenosis
CAD	coronary artery disease
CI-AKI	Contrast induced-Acute Kidney Injury
FFR	fractional flow reserve
GFR	glomerular filtration rate
LAD	left anterior descendent
PCI	percutaneous coronary interventions
RA	Rotational atherectomy
RCA	right coronary artery
SAVR	surgical aortic valve replacement
TAVI	trans-catheter aortic valve implantation
TLF	target lesion failure
ULM	unprotected left main
